# Cloth manipulation planning on basis of mesh representations with incomplete domain knowledge and voxel-to-mesh estimation

**DOI:** 10.3389/fnbot.2022.1045747

**Published:** 2023-01-05

**Authors:** Solvi Arnold, Daisuke Tanaka, Kimitoshi Yamazaki

**Affiliations:** Autonomous Intelligence and Systems Laboratory, Department of Mechanical Systems Engineering, Shinshu University, Nagano, Japan

**Keywords:** manipulation planning, cloth manipulation, deformable objects, neural networks, robotics, representation learning

## Abstract

Cloth manipulation is common in both housework and manufacturing. However, robotic cloth manipulation remains challenging, especially for less controlled and open-goal settings. We consider the problem of open-goal planning for robotic cloth manipulation, with focus on the roles of cloth representation and epistemic uncertainty. Core of our system is a neural network trained as a forward model of cloth behaviour under manipulation, with planning performed through backpropagation. We introduce a neural network-based routine for estimating mesh representations from voxel input, and perform planning in mesh format internally. We address the problem of planning with incomplete domain knowledge by introducing an explicit epistemic uncertainty penalty, using prediction divergence between two instances of the forward model network as a proxy of epistemic uncertainty. This allows us to avoid plans with high epistemic uncertainty during planning. Finally, we introduce logic for handling restriction of grasp points to a discrete set of candidates, in order to accommodate graspability constraints imposed by robotic hardware. We evaluate the system’s mesh estimation, prediction, and planning ability on simulated cloth for sequences of one to three manipulations. Comparative experiments confirm that planning on basis of estimated meshes improves accuracy compared to voxel-based planning, and that epistemic uncertainty avoidance improves performance under conditions of incomplete domain knowledge. Planning time cost is a few seconds. We additionally present qualitative results on robot hardware. Our results indicate that representation format and epistemic uncertainty are important factors to consider for open-goal cloth manipulation planning.

## Introduction

This work pursues versatile, open-goal planning abilities for robotic cloth manipulation (CM). Humans manipulate cloth with ease, but the actual planning process is notoriously hard to verbalise or formalise. Ubiquitous in household chores, CM is an important target for household robotics. However, its automation remains rare, even in controlled industrial settings. This low rate of automation is due to the fact that common manipulation methods assume object rigidity, an assumption cloth violates. To realise broadly applicable, generalised CM skills in robotic systems, we should address CM’s inherent complexities in a structured way.

**FIGURE 1:**
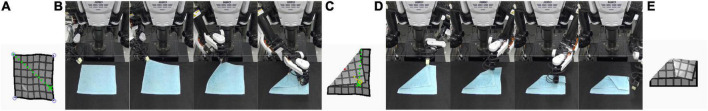
Execution of a two-step manipulation plan. **(A)** Estimate of initial state and first planned manipulation (dotted green line). **(B)** Execution of first manipulation. **(C)** Planned intermediate goal and second planned manipulation (dotted green line). **(D)** Execution of second manipulation. **(E)** Goal state.

Building on previous work, our approach emphasises flexibility and uncertainty. Our manipulation planning task assumes open start and goal shapes. While the task is not modelled after any specific household task, the requirements it presents capture some of the uncertainty and variability that household support robots will have to be able to deal with in order to operate effectively. Figure 1 shows an example plan being executed by a physical robot.

Some CM tasks can be solved with relative ease using specialised hardware. However, in a household setting it is preferable to solve many problems with few pieces of robotic hardware. We assume a dual-handed setup without specialisation to CM, consistent with the common conception of loosely humanoid support robots. We consider two arms to be the minimum for efficient CM, as many basic manipulations become significantly more difficult when performed single-handedly.

We also address the issue of planning with incomplete domain knowledge. Cloth items’ shape configurations have essentially infinite degrees of freedom. Explicitly representing item shapes at a decent level of precision yields high dimensional state spaces. Furthermore, high system flexibility requires a sufficiently broad action repertoire. Consequently, we have to operate in a sizable state-action space. Sampling such a large space exhaustively to generate training data is impractical and wasteful. Sampling only regions of interests is preferable, but necessitates a strategy for planning on basis of incomplete domain knowledge. The present work addresses this challenge using an explicit penalty to steer planning toward regions of low epistemic uncertainty.

## Related work

Here we discuss work related to ours by task setting or approach. As of yet there is no dominant strategy for robotic cloth manipulation. We discuss some of the strategies proposed so far, with a focus on flexibility and operation speed.

Fixed routines (Koishihara et al., 2017; Maitin-Shepard et al., 2010) and routines with simple branching (Yuba et al., 2017) have application in industrial settings and for particularly common manipulations, but lack the flexibility to accommodate variable start or goal states. Flexibility requires thinking ahead, which naturally suggests simulation-based solutions (Kita et al., 2014; Li et al., 2015). However, simulation-based approaches face challenges in achieving practical processing times.

### Reinforcement learning and forward models

One promising avenue for reconciling flexibility with practical operating speeds is seen in approaches employing neural networks (NNs). NNs have been applied in the context of CM for grasp point detection in a bed-making task (Seita et al., 2018) and prediction of forces exerted on human subjects in a dressing task (Erickson et al., 2018). NNs are also widely used in systems trained through Reinforcement Learning (RL), as have been applied to feedback-based folding (Petrík and Kyrki, 2019), and cloth smoothing (Wu et al., 2020). Methods using RL to learn from demonstrations have also been proposed over the past years, for, e.g., fabric smoothing (Seita et al., 2019) and dynamic manipulation (Jangir et al., 2020).

We employ NNs trained as forward models (FMs). We will refer to such NNs as FMNNs for short. The use of FMNNs for control tasks has been explored on various domains (Ebert et al., 2018; Henaff et al., 2017; Janner et al., 2019; Lesort et al., 2018; Wahlström et al., 2015), albeit in most cases on tasks of lower dimensionality than CM requires. The distinction between planning and model-driven control can be blurry, but control tasks typically assume shorter time-steps and fixed goals. The assumption of fixed or open goals, in particular, has consequences for the design of the system around the FM. FMs in the context of control tasks are often embedded in a RL context. A FMNN can replace the task environment in model-free RL to improve sample efficiency (Henaff et al., 2017; Wahlström et al., 2015), or be used as part of a model-based RL algorithm (Clavera et al., 2020). While such strategies are suitable for control tasks, planning as pursued here requires a higher level of flexibility. RL requires that a reward structure is set at training-time. Consequently, the majority of RL-based work assumes fixed goals. A rare exception is found in Jangir et al. (2020), where policy and reward functions are made conditional on a low-dimensional goal variable, making it possible to vary some aspects of the goal at run-time. However, whether this strategy could be extended to high-dimensional goal definitions is unclear.

Here, instead of learning a policy, we use the fact that NNs are differentiable to obtain gradients for the action inputs, allowing for fast action search on basis of a goal given at run-time. The differentiability of FMNNs has also been exploited in various fixed-goal RL approaches (Clavera et al., 2020; Henaff et al., 2017; Pereira et al., 2018). Our approach is similar to that of Henaff et al. (2017): backpropagation through the FMNN provides gradients for the action inputs, that guide action search toward high-quality actions. Also similar, albeit with a non-NN FM, is the approach of Watter et al. (2015). A second similarity with a subset of these systems is the use of latent state representations.

The task we target differs from typical RL tasks in robotics in a number of quantitative aspects. We noted the high dimensionality of adequate cloth shape representations. Ours is 3072D, which exceeds typical state dimensionalities in the current RL literature by a large margin. On the other hand, the number of steps we reason over is small, capping out at 3 in the present work where the control literature often considers tens of steps. However, the amount of time covered by a single pass through our FM is unusually long, spanning grasping, displacement, and release of the cloth item. The present work can in part be seen as an exploration of the effectivity of FMNNs in the high-dimensionality, long-timestep, short-rollout regime, which is an important domain for high-level planning.

### Forward models in cloth manipulation

For CM specifically, a few FMNN-based approaches have been proposed over the past years. Yang et al. (2017) report application to fine control in a CM task, albeit with fixed goal states. We previously proposed a system for open-goal, multi-step, dual-handed, FM-based CM planning, on basis of voxel input (Arnold and Yamazaki, 2019a; Tanaka et al., 2018). Kawaharazuka et al. (2019) apply a similar approach to string and cloth manipulation tasks involving momentum, using 2D images as input. Subsequently, Yan et al. (2020) proposed a method using a forward model in latent space, introducing contrastive learning to structure the latent space. States are represented as RGB images, and (single-step) manipulations are planned by random sampling. The method is applied to single-handed manipulation of ropes and cloth, with various goal shapes for rope. For complex goal shapes as we consider here, one-step greedy action generation is limiting. Hoque et al. (2020) propose a method using RGBD state representation and multi-step manipulation planning by CEM (Cross-Entropy Method), and apply it to cloth smoothing and basic single-handed folds.

Although spatial dimensionality and channel counts vary, all the above methods have in common that they use rasterisations (voxel volumes, images) of the workspace for state representation. These representations all have similar shortcomings. They are limiting in that they do not capture a topological understanding of the cloth’s shape configuration. One of our contributions is that we perform FM-based manipulation planning on basis of explicitly topological representations (mesh models). Consequently, our approach must incorporate mesh estimation.

### Mesh estimation

For mesh-based planning to be of use in robotic CM requires fast and robust mesh-estimation routines. Willimon et al. (2012) and Han et al. (2018) propose estimation routines for deformable objects, but the amount of deformation considered is limited. Sun et al. (2015) generate high quality 2.5D representations of wrinkled cloth in the context of a cloth flattening task, but here too the range of shapes considered is limited. Kita et al. (2014) and Li et al. (2014) consider more complex shapes, but their active observation strategies involve lifting and rotating the object, making these approaches unsuitable for our task scenario. We previously proposed an NN-based approach (Arnold and Yamazaki, 2019b). We integrate a variant of this approach in our planning system.

### Incomplete domain knowledge

We introduce measures to improve robustness under conditions of incomplete domain knowledge. Sampling the full state-action space to collect training data is costly and wasteful, as many state-action pairs are not particularly useful or interesting. This issue is compounded in dual-handed manipulation, due to increased dimensionality of the action space. Our solution for planning with incomplete domain knowledge employs dual instances of the FMNN. The use of network ensembles to boost performance or improve robustness has a long history (Granitto et al., 2005; Hansen and Salamon, 1990). Examples of ensemble methods are also found in the RL literature. Janner et al. (2019) and Clavera et al. (2020) use ensembles of FMs to counteract model bias. In these algorithms, trajectories for policy training are sampled from a randomly selected model instance from the ensemble. As noted above, our approach does not involve policy training, and consequently our use of multiple FMs differs from the RL setting. We will use a pair of FMNNs to calculate an explicit, differentiable proxy for epistemic uncertainty, and use this as a penalty to guide back-propagation-based plan search.

## Contributions

Here we discuss the present work’s main contributions.

### Cloth representation

In Arnold and Yamazaki (2019a), cloth states are represented in voxel format. Voxel representations are comparatively easy to obtain, invariant to differences in colour and patterning of cloth items, and robust against variable light conditions. Furthermore, they can be processed by conventional neural network architectures directly.

The voxel-to-voxel approach, as well as pixels-to-pixels approaches (Henaff et al., 2017; Hoque et al., 2020; Wahlström et al., 2015), are attractive for their conceptual simplicity: no special-purpose, engineered representation formats need to be considered. When latent representations are used, discovering a suitable representation format is left to the training process. However, the inherent ambiguity of a voxel representation limits how much fidelity can be attained. Topologically different cloth shapes can produce similar voxel representations. Consequently, voxel-based planning can produce outcomes that resemble the goal in voxel representation, yet diverge topologically. Additionally, for back-propagation-based planning, action gradients are not always informative if the states are given in image or voxel formats [as noted in (Yan et al., 2020) for the image case].

Bringing topological information into the planning process can be expected to improve fidelity. We employ deterministic and probabilistic mesh representations, with the latter allowing us to capture aleatoric uncertainty. This allows the system to pursue goal shapes with high accuracy where possible, and more loosely when uncertainty is high. Probabilistic meshes represent uncertainty at per-vertex, per-dimension granularity, so uncertainty can vary over different parts of the object. Furthermore, unlike voxel- and image-based state representations, the action gradients produced by mesh representations are guaranteed to be informative, as the error signal corresponds straightforwardly to mesh similarity.

A mesh-based approach is particularly advantageous in settings where significant occlusion occurs. Meshes allow leveraging of shape information from simulation data that is lost to occlusion in image-based state representation. Our mesh estimation system takes voxel input with occlusion present, but is trained to estimate complete meshes. Positions of occluded vertices cannot in general be determined exactly, but the positions of the visible vertices, as well as the occluded vertices’ invisibility itself, constrain the range of possible positions for occluded vertices. The system implicitly learns these constraints to locate occluded vertices, and because we let the mesh representation quantify positional uncertainty per vertex, the remaining uncertainty is quantified in a principled manner. Finally, the fact that goal states are specified in mesh form has the advantage that it allows us to specify target positions for occluded parts, which is not possible in image-based goal specification.

### Mesh estimation

To obtain mesh representations, we integrate the approach of Arnold and Yamazaki (2019b). Estimation consists of two steps. First we generate a probabilistic estimation using a simple NN architecture, and then we refine the estimate through an optimisation process incorporating prior knowledge of the cloth topology. We leverage the predictive abilities of the FMNN to resolve ambiguity of voxel representations in the estimation process. If a shape is the result of a manipulation generated by the system, we use the predicted outcome of the manipulation as additional prior knowledge to disambiguate the present state in the optimisation process. Hence shape information flows both ways between the mesh estimation system and planning system.

### Incomplete domain knowledge

For many task domains, only part of the state-action space is of practical interest. In a CM setup, many parametrisations of the manipulation format will make no useful change to many or most cloth shapes. Which parametrisations have useful effects will depend on the current shape of the cloth, so engineering the state-action space upfront to exclude uninteresting cases is infeasible. More fundamentally, it can be hard to formalise what is or is not useful. However, we *can* collect sets of relevant examples. A collection (dataset) of examples that samples only part of the full domain can be considered to (implicitly and fuzzily) define a “region of interest” (ROI) on that domain. A versatile planning system must be able to find good actions or action sequences on basis of experience that is restricted to this ROI. It should avoid uninteresting regions of the space, without requiring explicit definition of the ROI. However, FMNNs are vulnerable to incomplete domain knowledge, for two main reasons. First, for methods that rely on error gradients (as pursued here), gradients for unknown regions of the state-action space are unreliable, potentially causing plan search to spend time and computational resources chasing meaningless gradients without approaching the ROI. Secondly, the NN may spuriously predict outcomes resembling the goal state for state-action sequences leading through unknown regions of the domain. This can lead to generation of spurious plans that fool the system into expecting the goal state as outcome. This problem is closely related to the issue of adversarial examples (Goodfellow and Szegedy, 2014; Szegedy et al., 2013). In image recognition, input spaces (the space of all possible images of a given resolution) are so vast that training data can only sample a limited subspace of it (e.g., a set of real-world images). Such NNs are typically trained and tested on image sets that are assumed to sample roughly the same parts of the input space. Such NNs can and do produce unexpected classifications for unfamiliar inputs. Inputs intentionally designed to elicit erroneous classifications are known as “adversarial examples” (Szegedy et al., 2013), and are an active field of study. In neural network-based planning, training with datasets that only partially sample the input space can similarly lead to the existence of adversarial examples, in the form of what we might call “adversarial plans”, as we demonstrate in Arnold and Yamazaki (2019c).

The existence of adversarial plans can be a significant problem for these methods, and in some sense more so than in image recognition. Adversarial examples in image recognition are the product of “adversarial attacks”: attempts by an outside force to deceive the system. In a “white box” attack,^1^ the attacker may for example use a gradient descent approach to search the input space for images that deceive the NN into producing an erroneous classification (Athalye et al., 2018; Goodfellow and Szegedy, 2014; Kurakin et al., 2016). Now consider NN-based planning. These systems work by actively searching the NN’s input space for inputs that minimise a given loss. This means that if adversarial plans exist within the plan space, they appear as additional local optima, and plan search will pursue them just like it pursues valid plans. In other words, NN-based planning implements its own white box attack. We refer to the unintentional generation of adversarial plans as “self-deception”. Because the search process will approach adversarial plans just as it approaches valid plans, adversarial plans need not even be common in the plan space to pose a problem.

Ensuring that the NN is trained on complete domain knowledge precludes self-deception, but many domains are too large for exhaustive sampling to be practical, especially if data is to be sampled on robot hardware. This makes planning on basis of incomplete domain knowledge a problem of significant practical importance. We aim to realise versatile planning on basis of incomplete domain knowledge, using a dual-network approach for explicit epistemic uncertainty avoidance.

### Accommodation of grasp point detection routines

Reality imposes complex restrictions on which actions are physically possible or effective. In CM, we need to account for graspability. Grasp point detection is a challenging problem under active research (Li et al., 2019). Moreover, graspability depends on the specifics of the object and the robotic system. To accommodate this, we design our system to work with discrete sets of candidate grasp points provided by a subroutine. For our experiments, we assume non-specialised robot hardware equipped with gripper tips narrow enough to slide under a piece of cloth. Under these assumptions, cloth can be grasped easily by exposed corners, so we assume a simple candidate grasp point detection routine focusing on corners.

On a more abstract level, the assumption of a discrete set of grasp points produces a problem setting where each state presents with a variably sized set of continuous values for (part of) the action input (variably sized because the number of grasp point candidates varies with the cloth shape). This type of action space is rarely considered. Action domains of common RL benchmark tasks consist of a fixed discrete set of actions (e.g., moves in grid mazes), or a set of continuous action values (e.g., inverted pendulum balancing). Uncommonly, we see compound action domains consisting of discrete and continuous components (Henaff et al., 2017). Our case does not fit into any of these categories, but this type of action domain occurs naturally in tasks in which robots manipulate complex environments. Addressing this type of input regime in an FMNN-based planning system broadens applicability to practical tasks.

## System architecture

This section gives a global overview of the structure of the system, followed by detailed descriptions of its parts.

### Overview

Core of the system is an FMNN of the behaviour of a cloth item under manipulation. Given a cloth state and a manipulation, the FMNN predicts the resulting post-manipulation state. Manipulations are represented at coarse temporal granularity. A single manipulation grasps the cloth at one or two points, moves these points over a given distance, releases them, and waits for the cloth to settle in a stable state. This coarse granularity allows us to cover temporally extended manipulation sequences with a limited number of passes through the model.

The forward model operates on latent representations that encode mesh representations of cloth states. Meshes are obtained using an estimation routine taking voxel representations as input. The estimation routine consists of a voxel-to-mesh network (VtM net) that estimates a probabilistic mesh, and an optimisation process (“refinement”) that combines this probabilistic mesh with prior knowledge of the cloth topology and (if available) the preceding prediction of the current state, in order to generate a plausible deterministic mesh.

Plan generation takes the present state of the cloth and the intended goal state as input (both in mesh format). We find manipulation inputs that produce the latter from the former, by following manipulation input gradients obtained by backpropagation through the FMNN. Generation of multi-step plans (i.e., manipulation sequences) is achieved by recurrently chaining the FMNN.

Training the system involves two separate training processes. The first trains the FMNN, along with encoder and decoder modules for mapping full-size mesh representations to latent representations and vice versa. The second trains the VtM network to estimate probabilistic meshes from voxel representations. Both processes use the same dataset, which is generated in simulation. Data generation simulates the scenario of performing sequences of random manipulations on a square cloth the size of a hand towel, on a flat work surface.

### Representations

#### Cloth shape and position

**FIGURE 2:**
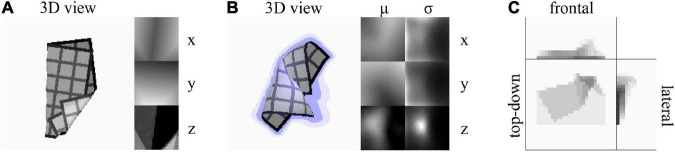
**(A)** A deterministic mesh representation (DMR). Left: Top-down 3D rendering of the mesh. Right: x, y, and z coordinates mapped as colour gradients in uv-space. Grey values for z-coordinates boosted for visibility. The grid texture on the 3D rendering is added for visualisation purpose only. The texture is not present in cloth observations and does not correspond to the mesh resolution. **(B)** A probabilistic mesh representation (PMR). Left: Top-down 3D rendering of the μ-component of the mesh. Lilac shading around the cloth indicates the σ_*x*_ and σ_*y*_ components, and lilac cast on the cloth indicates the σ_*z*_ component. Right: μ_*x*_, μ_*y*_, μ_*z*_, σ_*x*_, σ_*y*_, σ_*z*_ components mapped as colour gradients in uv-space. Grey values for μ_*z*_ and σ_*z*_ boosted for visibility. **(C)** A voxel representation. Colour indicates mean voxel value over each voxel column parallel to the viewing angle.

We use three shape representations: voxel, deterministic mesh, and probabilistic mesh. We abbreviate the latter two as DMR and PMR, respectively. Voxel representations are binary matrices of resolution 32 × 32 × 16. DMRs are real-valued matrices of size 32 × 32 × 3, assigning (x, y, z) coordinates to each vertex of a 32 × 32 mesh topology. PMRs resemble their deterministic counterpart, but represent each vertex position as a multivariate normal distribution. They are real-valued matrices of size 32 × 32 × 6, with each 1 × 1 × 6 subvolume containing a set of means (μ_*x*_, μ_*y*_, μ_*z*_) and standard deviations (σ_*x*_, σ_*y*_, σ_*z*_) probabilistically describing the position of a single vertex in the mesh. The σ values define the diagonal of the multivariate normal distribution’s covariance matrix. A more complete representation would specify the full covariance matrix, but we wish to constrain the number of outputs to be learned. Example visualisations of a DMR, PMR, and a voxel representation are given in Figure 2.

Cloth shapes are always centred in the XY plane. We find the cloth centre point by projection onto the XY plane to obtain a 2D image, and calculating coordinate averages over all pixels corresponding to the cloth. Centre point coordinates are stored separately, and the cloth is then shifted in the XY plane to bring the centre point to (0, 0). By splitting states into a shape component and a position component, we obtain position-invariant shape representations, allowing generalisation over positions. Position information can be important when, e.g., planning under restriction of a limited work surface or motion range. We do not consider such constraints here, but we note that by adding the predicted offsets to predicted vertex coordinates, we can recover a mesh prediction that is located in the workspace, and define additional planning losses thereon as suits a given scenario.

#### Manipulation

Manipulations are real-valued vectors of length six. The first four values define two grasp points, $g_{1}^{G}$ and $g_{2}^{G}$, using geodesic coordinates (*u, v*), with the cloth running from (-1, -1) to (1, 1). The second grasp point can take null values, indicating a single-handed manipulation. The last two values in the vector are a displacement vector $\vec{d}$ given in 2D Cartesian coordinates.

Given a cloth state, this representation determines grasp point trajectories as follows. Using the mesh representation of the cloth state, we map geodesic grasp points $g_{i}^{G}$ to Cartesian grasp points $g_{i}^{C}$. For two-handed grasps, we then compute point *p* as


(1) $$\begin{array}{c} \text{p}=\frac{{\text{g}}_{1}^{C}+{\text{g}}_{2}^{C}}{2}+\frac{\vec{\text{d}}}{2}=\frac{{\text{g}}_{1}^{C}+{\text{g}}_{2}^{C}+\vec{\text{d}}}{2}. \end{array}$$

**FIGURE 3:**
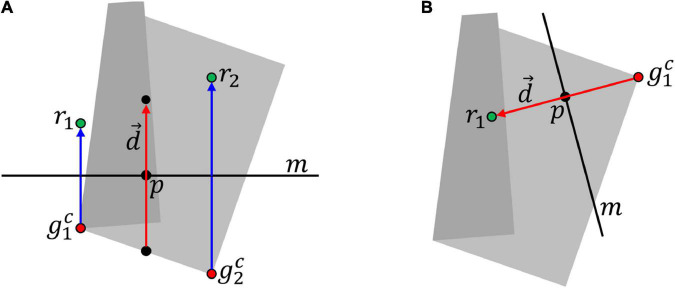
Calculation of trajectories. **(A)** Dual-handed case. Trajectories (blue lines) and release points (*r*_1_, *r*_2_) are calculated from grasp points ($g_{1}^{C},g_{2}^{C})$ and displacement vector $\vec{d}$. In this case, $\vec{d}$ describes the displacement of the point in between the two grasp points. **(B)** Single-handed case. In this case, $\vec{d}$ describes the displacement of the sole grasp point $g_{1}^{C}$, so release point *r*_*1*_ is found by adding $\vec{d}$ to $g_{1}^{C}$. The grey shape in the background is the 2D projection of the cloth.

Let *m* be a line through *p* perpendicular to $\vec{d}$. The *x* and *y* coordinates for Cartesian release points *r_i_* are found by mirroring $g_{i}^{C}$ over line *m* on the XY-plane. For single-handed grasps, the *x* and *y* coordinate of the single release point *r*_1_ is given by $g_{1}^{C}+\vec{d}$. Figure 3 illustrates the calculation for a dual- and single-handed manipulation.

The z coordinate for *r_i_* is given by $g_{i}^{C}.z+min\left(z_{drop},k/2\right)$, where $g_{i}^{C}.z$ is the z-coordinate of point $g_{i}^{C}$ and *k* is the distance between $g_{i}^{C}$ and *r_i_* in the XY-plane. We find the unique circle *c* centred at height $g_{i}^{C}.z$, perpendicular to the XY plane, and passing through $g_{i}^{C}$ and *r_i_*. The shortest arc segment of *c* connecting $g_{i}^{C}$ and *r_i_* defines the trajectory for point *i*. System parameter *z*_*drop*_ controls the maximum height (relative to the grasp point) from which the cloth is released. For trajectories whose apex is less than *z*_*drop*_ above $g_{i}^{C}.z$, the drop height instead equals the height of the apex.

The manipulation format improves on that of Arnold and Yamazaki (2019a) in terms of flexibility and similarity to human cloth manipulation. Trajectories produced by this format rarely cause the cloth to slide over the work surface, and better resemble human cloth manipulation. Consider for example the manipulation that folds a flaring skirt in two over its vertical axis, grasping it at the waistband and lower edge. The grasp points will lie at different distances from the fold line (*m*), and the grasp points will be moved by different distances in the same direction in the XY plane. The new format concisely represents such folds. This format produces trajectories resembling those considered in Van den Berg et al. (2010), although our motion in the z dimension is rounded instead of triangular.

Using geodesic coordinates for grasp points is advantageous, because geodesic space directly corresponds to the cloth surface, whereas most points in the Cartesian workspace do not correspond to positions on the cloth surface. Defining grasp points in Cartesian space would thus inflate the search space. Geodesic coordinates of all mesh vertices are known and fixed, so given a DMR of the cloth, conversion between Cartesian and geodesic grasp point coordinates is trivial. For executing manipulations, coordinates are converted into Cartesian form.

### Grasp point detection

To constrain grasp points to actually graspable points of potential interest, we introduce a simple detection routine. We first project the cloth onto the XY plane to obtain its top-down silhouette. We then use corner detection (Shi and Tomasi, 1994) to obtain the corner points of the silhouette, and discard concave corners. The resulting set of convex corners comprises the graspable point set for the cloth shape. These points will generally be easy to grasp with a variety of robot hands by horizontally approaching the cloth along the table surface.

As noted above, grasp points are represented as geodesic (*u, v*) coordinates in the manipulation definition. Detected grasp points are converted to geodesic space by assigning the (*u, v*) coordinates of the cloth vertex nearest to the grasp point.

Our choice of grasp point candidates provides sufficient shape variation to produce a challenging task domain, while also facilitating hardware experiments. However, the system does not depend on the details of the routine, so different detection routines can be substituted here to suit different robot platforms or tasks. While we use an image-like cloth representation for grasp point detection here, a mesh estimation of the current cloth shape exists whenever grasp point detection is performed. Hence more advanced detection routines could exploit the mesh estimate in addition to image data to find different or additional graspable points (e.g., exposed corners inside the cloth silhouette).

### Data collection

We collect manipulation examples using the ARCSim simulator (Narain et al., 2012; Narain et al., 2013). We construct a scene containing a square cloth laid out flat on a level work surface. Manipulation sequences of length three are applied, and the resulting cloth states are stored. We generate single- and dual-handed manipulation examples in a ratio of 1:2. Manipulations are randomly generated under the following restrictions.

1. Graspability restriction. Grasp points are restricted to the set generated by the detection routine described above. Assuming the routine accurately captures the grasping abilities of the target platform, this restriction allows us to constrain data collection to pertinent examples.

2. Fold restriction. Manipulations that have little or no effect on the cloth shape are of limited interest. To avoid (a large proportion of) such cases we restrict displacement vectors as follows. We define reference point *q* as $\left(g_{1}^{C}+g_{2}^{C}\right)/2+0.8\vec{d}$ for dual-handed manipulations or $g_{1}^{C}+0.8\vec{d}$ for one-handed manipulations. Only manipulations for which *q* lies on the cloth (i.e., falls within the projection of the cloth) are included in the dataset. Preliminary experimentation indicated that the majority of manipulations that meet this condition produce folds.

3. Displacement distance restriction. Manipulations with very short displacement distances tends to make no appreciable change to the cloth shape, because such folds undo themselves during shape stabilisation. We enforce that the length of displacement vector $\vec{d}$ should be at least 0.35 times the length of the cloth, and let actions with displacement vectors lengths below this threshold represent null-manipulations instead.

These restrictions help to produce a dataset that contains a higher proportion of interesting samples, with fewer examples that are overly crumpled or simple position shifts. This data is more representative of human cloth folding behaviour than unrestricted sampling, and lets us zoom in on manipulations of higher practical value. However, data collected under these restrictions amounts to incomplete domain knowledge, necessitating countermeasures.

ARCSim employs adaptive remeshing, changing the mesh topology dynamically to best express the shapes of cloth items as they deform. Our system assumes a fixed topology. We convert meshes to our fixed topology via interpolation in post-processing.

We generate a dataset of 3693 sequences, containing a total of 11079 manipulation examples. The dataset is split into training, test, and validation sets of 3493, 100, and 100 sequences, respectively.

### Probabilistic EM*D net

This NN forms the core of the system, and extends our previously proposed architecture. We focus on the points of improvement, and refer to Arnold and Yamazaki (2019a) for any aspects not covered. The pEM*D net consists of an encoder module E, a manipulation module M (the FMNN), and a decoder module D, with functions defined as follows. Encoder net E maps DMR *s_i_* to its latent representation (LR) ${\hat{c}}_{i}$:


(2) $$\begin{array}{c} \text{E}\left({\text{s}}_{i}\right)={\hat{\text{c}}}_{i}. \end{array}$$

LRs are real-valued vectors of length 512. Manipulation net M maps a tuple consisting of LR *c*_*i*_, memory trace *t_i_*, and manipulation *m*_*i*_ to a LR ${\hat{c}}_{i+1}$ of the predicted outcome shape, a new memory trace *t*_*i+1*_, and a prediction of centre point shift $△{\hat{a}}_{i+1}$:


(3) $$\begin{array}{c} \text{M}\left({\hat{\text{c}}}_{i},{\text{t}}_{i},{\text{m}}_{i}^{{}^{\prime }}\right)=\left({\hat{\text{c}}}_{i+1},{\text{t}}_{i+1},△{\hat{\text{a}}}_{i+1}\right), \end{array}$$

where $m_{i}^{\prime }$ is *m*_*i*_ with its grasp points shifted by −*a*_*i*_, with *a*_*i*_ denoting the centre point for *s*_*i*_. As centre point movement is relative to the pre-manipulation cloth state, no centre point location needs to be included in M’s input. Memory traces, too, are real-valued vectors of length 512. We let *t*_*0*_ be the zero vector. Decoder net D maps a LR ${\hat{c}}_{i}$ of a cloth state to its PMR ${\hat{s}}_{i}$:


(4) $$\begin{array}{c} \text{D}\left({\hat{\text{c}}}_{i}\right)={\hat{\text{s}}}_{i}. \end{array}$$

Predictions for multi-step plans are generated by propagating through M recurrently, with different manipulation inputs at every pass. For notational convenience we define the following shorthand:


(5) $$\begin{array}{c} {\text{EM}}^{n}\text{D}\left({\text{s}}_{i},m\right)=\text{D}\left({\hat{\text{c}}}_{i+n}\right), \end{array}$$

$${\hat{\text{c}}}_{i+1}=\pi_{1}\left(\text{M}\left({\hat{\text{c}}}_{i},{\text{t}}_{i},{\text{m}}_{i}^{{}^{\prime }}\right)\right),$$

where *m* is a manipulation sequence of length *n*, and π_*i*_ is the *i^th^* projection (i.e., π_*i*_ selects the *i^th^* element of its argument tuple).

**FIGURE 4:**
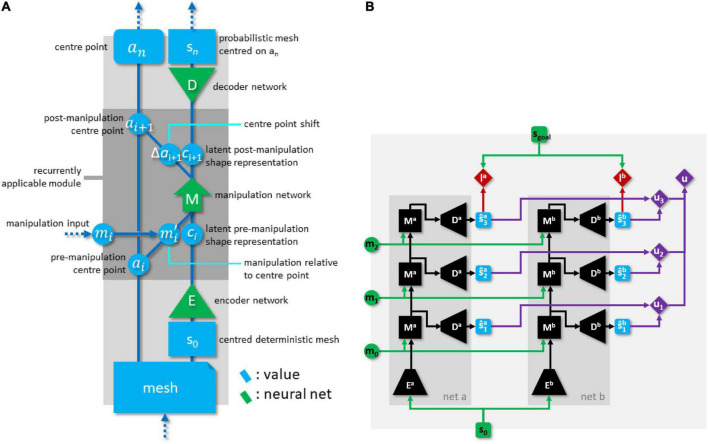
**(A)** Global structure of the pEM*D net. **(B)** Dual network architecture with explicit epistemic uncertainty penalty, rolled out for 3-step plans. Green items indicate network inputs and (externally supplied) goal state. Black items are neural network modules. Blue items are state predictions. Red items are losses. Purple items comprise the epistemic uncertainty penalty calculation.

Size parameters of the pEM*D net are given in Table 1, and its structure is illustrated in Figure 4A.

**TABLE 1:** pEM*D network architecture parameters.

Module	E	M	D
Input	32 × 32 × 3 (*s*_*i*_)	512 (${\hat{c}}_{i}$) + 512 (*t*_*i*_) + 6 ($m_{i}^{\prime }$)	512 (${\hat{c}}_{in}$)
Neuron layers	4	16	4
Hidden layer sizes	2048, 1024	1024 + 6 ($m_{i}^{\prime }$)	1024, 2048
Output	512 (${\hat{c}}_{i}$)	512 (${\hat{c}}_{i+1}$) + 512 (*t*_*i+1*_) + 2 ($△{\hat{a}}_{i+1}$)	32 × 32 × 6 (${\hat{s}}_{in}$)

Connectivity in the M module mixes regular propagation, residual propagation, and skip connections. Propagation from the first 512 neurons in layer *i* to the first 512 neurons in layer *i+1* is residual (i.e., values from the first 512 neurons are copied to the next layer, followed by regular fully connected propagation). Each layer *i* for which *i* is even and *0 < i < 14* has skip connections projecting into the first 512 neurons of layer *i+2*. This heterogenous connectivity is based on the idea of letting M iteratively transform the state representation passing through its residually connected channel.

A simple local response normalisation is applied over the activation vector in all layers except for the hidden layers of the M module and the output layer of the D module:


(6) $$\begin{array}{c} {\text{act}}_{o}=\frac{{\text{act}}_{i}}{\sqrt{1{\left|{\text{act}}_{i}\right|}_{2}^{2}}}, \end{array}$$

where *act*_*i*_ and *act*_*o*_ are ingoing and outgoing activation, and |⋅|_2_ is the L2 norm.

Hidden layers use the hyperbolic tangent activation function. In the decoder module, output neurons representing μ components use the identity function, and output neurons representing σ components use


(7) $$\begin{array}{c} {\text{act}}_{o}={\text{e}}^{{\mathrm{act}}_{i}}+0.01, \end{array}$$

where *act*_*i*_ and *act*_*o*_ are ingoing and outgoing activation. This function has a minimum output value of 0.01, which helps stabilise training, as likelihood loss can fluctuate dramatically when σ grows too small. With regards to its probabilistic output, the pEM*D net follows Mixture Density Networks (Bishop, 1994), although we use only one distribution per variable.

Given the centre point of the initial state, we can find predicted cloth locations by adding the $△{\hat{a}}_{i}$ output of subsequent iterations through the M module:


(8) $$\begin{array}{c} {\hat{\text{a}}}_{i}={\text{a}}_{0}\sum_{j=1}^{i}△{\hat{\text{a}}}_{j}. \end{array}$$

Values ${\hat{a}}_{i}$ could be used to define additional planning losses to constrain plan search.

### Probabilistic EM*D net training

We train modules E, M, and D end-to-end, thereby forcing E and D to learn a latent representation format that facilitates application of manipulations by M. The compound net is trained on batches of 64 manipulation sequences of random length between 1 and 3 steps. We use two losses, one measuring shape prediction accuracy (*loss*_*s*_) and one measuring accuracy of the predicted position change (*loss*_*p*_). *Loss*_*s*_ for a single example is defined as follows:


(9) $$\begin{array}{c} {\text{loss}}_{s}=\sum_{i=1}^{N_{\mathrm{steps}}}\text{NLL}\left({\text{s}}_{i},{\hat{\text{s}}}_{i}\right), \end{array}$$

where NLL is shorthand for negative loss-likelihood and *N*_*steps*_ is the length of the sequence. *Loss*_*p*_ is the MSE of the predicted offsets w.r.t. the actual offsets.

In contrast to typical recurrent neural network training, this loss sets a target for each pass through the M module. The *i*^th^ pass produces ${\hat{c}}_{i}$, which D decodes to ${\hat{s}}_{i}$, which is compared against ground truth *s_i_*. Hence, training constrains the output for each pass to the same extent as in non-recurrent architectures. This simplifies training.

Training computes connection weight gradients for both losses, and combines the losses by summing their signs at the connection level. This allows for combination of losses without adding hyperparameters for weight balancing. Weights are updated using the SignSGD update rule (Bernstein et al., 2018), so gradient magnitudes are discarded. We previously found that SignSGD is more effective than standard SGD for training this type of architecture (Arnold and Yamazaki, 2019a). The learning rate is initialised to 5 × 10^–5^. Every 10000 iterations, we measure prediction accuracy on the validation set, and reduce the learning rate by a factor 2 when there has been no improvement in 50K iterations. Training runs for 1.6M iterations. We use the nets with the best prediction accuracy on the validation set for further evaluation.

**FIGURE 5:**
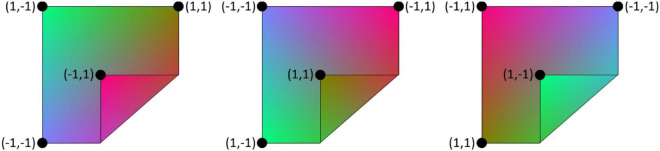
Equivalent mesh representations of a cloth shape. Geodesic (uv) coordinates are shown for the cloth corners and a gradient texture is added to visualise geodesic coordinates over the cloth surface. The representations differ in how they map geodesic space to Cartesian space, but represent the same shape. For every possible shape configuration of a square cloth, there are eight equivalent mesh representations.

Mesh vertex coordinate input is presented in a normalised form where the cloth in its fully spread, axis-aligned state runs from (-0.7, -0.7) to (0.7, 0.7) in the XY plane. Training employs five types of data augmentation. (1) Rotation and mirroring of manipulation sequences. (2) Introduction of non-manipulation examples. An example is converted into a non-manipulation example by replacing the action with a null-manipulation, and replacing the post-manipulation state with a copy of the pre-manipulation state. This is to learn the non-effect of null-manipulations. Recall that null-manipulations are represented as any action with a displacement vector shorter than 0.35 times the length of the side of the cloth. (3) Grasp point swaps. Grasp point order is immaterial, so swapping the grasp points results in a different representation of the same manipulation. (4) Addition of Gaussian noise with σ = 0.025 to vertex coordinates. (5) Conversion of input and target DMRs to any of their equivalent representations. For a square cloth, each possible shape has eight valid mesh representations, differing in how Cartesian coordinates are assigned to geodesic coordinates. We refer to this as *Cartesian equivalence*. Figure 5 illustrates the concept. In data augmentation we convert states into randomly selected equivalents, and convert manipulation inputs accordingly.

Module M expects two grasp points for its manipulation input. For single-handed manipulations, we input the same grasp point on both inputs.

### Voxel-to-mesh conversion

The second NN converts voxel representations to mesh representations (Arnold and Yamazaki, 2019b). We refer to this network as the Voxel-to-Mesh (VtM) net. This is a 7-layer MLP with input and output layer sizes matched to the voxel representation size and PMR size, respectively, and hidden layer size 4096 throughout. Input is given as a voxel representation with the cloth centred in the voxel space. We use a normalised coordinate system in which the voxel viewport runs from (-1, -1, 0) to (1, 1, 0.125). As for pEM*D input, the fully spread, axis-aligned cloth runs from (-0.7, -0.7) to (0.7, 0.7). This scaling is chosen so that any centred cloth shape fits within the viewport in full. Resolution is four times finer on the z-axis than on the other axes, to account for the fact that stable cloth shapes present detail such as wrinkles and layering on the z-axis, while spatial extension on the z-axis is limited.

We denote the VtM net’s functionality as


(10) $$\begin{array}{c} \text{VtM}\left({\text{s}}_{i}^{v}\right)={\dot{\text{s}}}_{i}^{p}, \end{array}$$

where $s_{i}^{v}$ is the voxel representation and ${\dot{s}}_{i}^{p}$ a probabilistic mesh estimate of the state at manipulation step *i*.

VtM produces PMRs, but for the planning procedure, we need a DMR, as deterministic graspable points can only be computed for DMRs. After estimating ${\dot{s}}_{i}^{p}$, we search for DMR ${\dot{s}}_{i}^{d}$ that is most (or at least highly) plausible w.r.t. ${\dot{s}}_{i}^{p}$. We refer to this procedure as *refinement* and denote it as


(11) $$\begin{array}{c} \text{R}\left({\dot{\text{s}}}_{i}^{p},{\tilde{\text{s}}}_{i}^{d}\right)={\dot{\text{s}}}_{i}^{d}, \end{array}$$

where ${\tilde{s}}_{i}^{d}$ is a DMR used to initialise the search process. We use the μ component of a previously obtained prediction for the current state as ${\tilde{s}}_{i}^{d}$ when available, or simply use the μ component of ${\dot{s}}_{i}^{p}$ otherwise. The DMR ${\dot{s}}_{i}^{d}$ is initialised to ${\tilde{s}}_{i}^{d}$, and iteratively refined to minimise the following loss measures:

• loss_nll_: Negative log-likelihood of ${\dot{s}}_{i}^{d}$ w.r.t. ${\dot{s}}_{i}^{p}$.
• loss_spring_: Spring energy.
• loss_up_: upward bias loss.

To compute loss_*spring*_, we define a set of springs between the vertices of the mesh, following a spring pattern common to cloth simulation [see e.g., (Choi and Ko, 2002)]. Let *k* be the distance between orthogonally neighbouring vertices. A vertex at indices (*u, v*) in the cloth connects to neighbour vertices (*u, v* ± *k*) and (*u* ± *k, v*) (“stretch” springs), (*u* ± *k, v* ± *k*) (“shear” springs), and (*u, v* ± 2*k*) and (*u* ± 2*k, v*) (“bend” springs), insofar these vertices exist. Spring energy loss is then calculated as


(12) $$\begin{array}{c} {\text{loss}}_{\mathrm{spring}}=\sum_{i=0}^{N_{\mathrm{springs}}}{\left({\text{l}}_{i}-{\text{r}}_{i}\right)}^{2}, \end{array}$$

where *l*_*i*_ is the current length of the spring, *r*_*i*_ the resting length of the spring, and *N*_*springs*_ the total number of springs.

Loss_*up*_ biases optimisation against adjusting vertex positions downward, because this can push vertices into the working surface. Upward bias ensures that wrinkles produced by optimisation form in upward direction. This bias loss is computed as the mean over $max\left(0,{\dot{s}}_{i}^{p}.\mu_{z}-{\dot{s}}_{i}^{d}.z\right)$, where ${\dot{s}}_{i}^{p}.\mu_{z}$ and ${\dot{s}}_{i}^{d}.z$ are the μ_*z*_ and *z* components of ${\dot{s}}_{i}^{p}$ and ${\dot{s}}_{i}^{d}$ and subtraction and *max* operate element-wise. Spring loss and upward bias loss are multiplied by 5000 and 1000, respectively, to be on the same order of magnitude as loss_*nll*_. We update vertex positions using SignSGD on the compound loss, with an update step of 0.001. Optimisation runs until loss stabilises or 300 iterations have passed.

Details such as small creases are hard to estimate from voxel representations. The PMR produced by VtM tends to miss such detail in its μ component. Where small creases or other details have disappeared, spring lengths computed over the μ component come out short, and σ values are elevated. Higher σ values allow the optimisation step to move vertices further from their μ positions with smaller impact on loss_*nll*_. Consequently, optimisation tends to fill in missing detail by moving vertices around so as to recover correct spring lengths, within the leeway provided by the locally elevated σ values. The resulting DMRs are thus more realistic cloth shapes than would be obtained by simply taking the μ component of the PMR. This realism is important for subsequent processing, as the pEM*D net is trained on deterministic mesh data from the simulation. Skipping refinement was observed to significantly harm planning performance. However, refinement does not necessarily improve accuracy w.r.t. the ground truth compared to the μ component of the VtM estimate (a missing crease may yield a smaller error than a crease recovered at a slight offset).

The choice to use an uncoloured voxel representation as input for mesh estimation is motivated by the fact that this representation naturally generalises over variations in colour, texture, and lighting conditions, whereas coloured representations require extensive domain randomisation for generalisation (Wu et al., 2020; Lips et al., 2022). Furthermore, in preliminary experimentation, voxel input produced better estimation accuracy than depth images of the same resolution.

### Voxel-to-mesh training

The VtM net is trained on the shape data from the dataset. Meshes are converted to voxel representation for input by setting all voxels that contain at least one vertex to 1 and all other voxels to 0. Before voxelisation, we double the mesh resolution by interpolation, duplicate the resulting set of vertices and apply Gaussian noise to vertex coordinates with σ = 0.025, to promote generalisation. Noise is absent in the ground truth mesh, so the net also learns to denoise its input. We apply artificial self-occlusion by setting to 1 all voxel for which an occupied voxel with the same *x* and *y* index and a larger *z* index exists, approximating the self-occlusion that occurs for a single top-down camera viewpoint. We apply data augmentation by mirroring and random rotation around the Z axis. Rotation in particular proved crucial to avoid excessive overfitting.

VtM net training must account for Cartesian equivalence. As each shape has eight equivalent mesh representations, there exist eight correct answers for each valid input to the VtM net. We found no principled way of designating any one answer as canonical. Using the meshes as-is as training targets leads to failure to train, because augmentation and repeated manipulation render the original assignment of Cartesian coordinates to geodesic coordinates opaque. We allow all assignments by training with the following loss:


(13) $$\begin{array}{c} {\text{loss}}_{\mathrm{VtM}}=\text{min}\;\left\{\text{NLL}\left({\text{s}}^{j},{\dot{\text{s}}}_{i}^{p}\right)|\text{j}\in \left[0,\ldots ,7\right]\right\}, \end{array}$$

where *s^j^* is the *j*^th^ equivalent assignment in an arbitrary ordering of the ground truth’s equivalent assignments. Note that the *min* operator here allows the training process flexibility to determine which one of the correct answers to pursue for each input. Training uses the signSGD update rule with automated learning rate reduction as in pEM*D training, and is terminated when the learning rate drops below 10^−7^.

## Planning logic

Next we explain the planning algorithm. We perform planning through backpropagation, and address the problem of incomplete domain knowledge by introducing an epistemic uncertainty penalty term in the loss function minimised by the planning procedure.

### Planning algorithm

Manipulation planning starts from a voxel representation of the current cloth state. When planning on basis of simulation data, we convert the DMR provided by the simulator to voxel format to emulate the real-world case. The voxel representation $s_{0}^{v}$ is converted to PMR ${\dot{s}}_{0}^{p}$ using the VtM net. Then we derive DMR ${\dot{s}}_{0}^{d}$ from ${\dot{s}}_{0}^{p}$ with the refinement routine described in Section 4.7, using the μ component of ${\dot{s}}_{0}^{p}$ as initialisation value. We apply the grasp point detection routine on this DMR to obtain the set of graspable points for the current shape. On basis of ${\dot{s}}_{0}^{d}$ and DMR s* representing the goal state, we generate a manipulation plan. We assume the number of steps *n* to be given and ≤ 3. Generation of a plan of length *n* is then performed as follows:

1. Initialise a random plan *m* = < *m_*i*_, …, m_*i*+*n–1*_* >
2. Compute the planning loss (detailed below) for *m*.
3. If *n*_*iterations*_ iterations have passed, return the plan with the lowest planning loss seen so far.
4. If the loss score has not improved for 5 steps, return to step 1.
5. Obtain loss gradients w.r.t. the manipulation inputs by means of backpropagation of the planning loss.
6. Adjust *m* in accordance with these gradients using the iRprop- update rule (Igel and Hüsken, 2000), and return to step 2.

The number of search iterations is set in consideration of the plan length: *n*_*iterations*_ = *25 + 25n*. Hyperparameters for iRprop- were set as follows: η*^+^* = 1.2, η*^–^* = 0.5, Δ*_*min*_* = 10^–3^, Δ*_*max*_* = 0.05. Update rates are initialised to 0.05. We run 256 planning instances in parallel, and adopt the plan with the lowest residual plan loss seen over the course of the planning processes in all instances.

The planning logic must account for the unusual action format. Recall that we have a variably sized, discrete set of real-valued grasp point candidates for the current state. In step (1), grasp points for the first manipulation in the plan are picked at random from the grasp point set of the current state. These grasp points are not updated in step (6). Candidate grasp points for the first manipulation are known, so there is no need to search the input space aside from this discrete set. For manipulations beyond the first, we only have (continually changing) probabilistic mesh predictions. This makes it hard to derive candidate grasp points, so we leave it up to the search process to figure out where viable grasp points are likely to appear. Non-first grasp point positions need not be exact: we re-run planning after each manipulation in order to incorporate the new shape observation, so non-first manipulations are never executed.

Different Cartesian-equivalent mesh representations of the same shape may elicit slightly different plans. We compute all equivalents of the current (${\dot{s}}_{i}^{d}$) and goal DMR (s*), and assign one eighth of the planning instances to each. For each non-first planning process in a trial, we initialise one instance per equivalent with a modified version of the previously generated plan. This modification drops the first manipulation (as it has just been performed), and snaps the grasp points of the new first manipulation to the nearest points in the new set of candidate grasp points.

Once a plan is generated, we check whether the system in fact expects the plan to produce a better approximation of the goal than the (estimated) current state provides. If it does not, we replace the next manipulation with a null manipulation.

The first manipulation of the generated plan is executed in simulation or hardware. After execution, we observe the resulting cloth state as a voxel representation, and store the outcome. If the plan length was 1, we terminate the interaction. Otherwise, we repeat the planning process with plan length *n* reduced by 1.

For subsequent steps of a multi-step manipulation process, we carry over some information from the preceding step. $EM^{1}D\left({\dot{s}}_{i}^{d},m_{i}\right)$ gives a probabilistic expectation ${\hat{s}}_{i+1}$ of the outcome of the first manipulation in the generated plan. For the subsequent plan generation process, we let ${\dot{s}}_{i+1}^{d}=R\left(VtM\left(s_{i+1}^{v}\right),{\hat{s}}_{i+1}.\mu \right)$. By using the μ component of the prediction for the new state as initial mesh for refinement, we aim to disambiguate states that are ambiguous in voxel representation. This biases refinement toward interpretations of the voxel input that are consistent with the predicted outcome of the preceding manipulation.

A second way in which we carry through information from preceding steps is via memory trace *t*_*i*_. At each subsequent plan generation process in a trial, we use memory trace *t*_*i+1*_ obtained through $M\left(E\left({\dot{s}}_{i}^{d}\right),t_{i},m_{i}^{\prime }\right)$ as the memory trace input for the first instance of *M*. This serves to allow preceding manipulations to influence plan generation. *t*_*i+1*_ is recomputed for all Cartesian equivalences by performing a single forward propagation through pEM*D.

### Planning loss and epistemic uncertainty avoidance

Next we address the problem of planning with incomplete domain knowledge. The aim here is to constrain plan search to the domain covered by the training data (the ROI), because only predictions within this domain are reliable. This is a problem of epistemic uncertainty avoidance. In contrast to aleatoric uncertainty, NNs cannot quantify epistemic uncertainty about their outputs, so a different approach is necessary. We introduce a dual network strategy, based on the following principle: two networks trained independently on the same training data will produce similar predictions for inputs in the domain covered by that training data. However, for inputs outside this domain, predictions are not constrained to be similar between the networks, and will diverge. Hence, we can use the discrepancy between predictions from two independently trained networks as a *proxy* of whether an input (i.e., plan) lies within the known (trained) domain or not.

We distinguish implicit and explicit avoidance. Implicit avoidance is obtained by simply combining prediction losses from the two independently trained nets into a single planning loss. There should be fewer plans that deceive both nets into predicting an outcome close to the goal than plans that deceive just one net into doing so. Therefore, few plans leading through unknown regions of the state-action space would produce a low combined prediction loss.

This implicit strategy relies on the fact that discrepancy between predictions will raise the combined prediction loss. However, various other factors affect the combined prediction loss, making this a poor proxy of epistemic uncertainty as such. Given two nets, we can also calculate an explicit measure of prediction discrepancy. This allows us to also take into account prediction discrepancy on the non-final states of a manipulation plan. Plans for which both nets predict similar outcomes will still be unreliable if predictions for intermediate states diverge.

Hence, we introduce an epistemic uncertainty penalty term *u* in the planning loss function. Planning loss for dual-net planning with *net*_*a*_ net and *net*_*b*_ is defined as


(14) $$\begin{array}{c} \begin{array}{l} loss_{dual}=\frac{NLL\left(w\left(s^{*}\right),w\left({\hat{s}}_{n}^{a}\right)\right)-low_{n}^{a}}{high_{n}^{a}-low_{n}^{a}}+\frac{NLL\left(w\left(s^{*}\right),w\left({\hat{s}}_{n}^{b}\right)\right)-low_{n}^{b}}{high_{n}^{b}-low_{n}^{b}}\\ \;+\alpha \cdot u, \end{array} \end{array}$$

where s* is the goal state, ${\hat{s}}_{n}^{j}$ is *net*_*j*_’s predicted outcome for the current plan, $high_{n}^{j}$ and $low_{n}^{j}$ are reference values for normalising the losses from each net, α is a fixed weight, and *u* is the epistemic uncertainty penalty for the current plan. We refer to the first two terms as accuracy terms and to the last term as the epistemic uncertainty term. Function *w* is a simple whitening operation, rescaling input states such that vertices have a mean of 0 and a standard deviation of 1 for each Cartesian dimension. For PMRs this is over the μ elements, with σ values rescaled accordingly. This eliminates the effect of the overall size of a cloth shape on the loss. Figure 4B illustrates information flow in the dual-net compound architecture.

Normalising the accuracy terms is not strictly necessary, but simplifies balancing with the uncertainty term. It also avoids the need to retune α when changing networks, allowing for fairer comparison experiments. Normalisation is only meaningful when combining multiple loss terms, as our planning algorithm employs iRprop-, which uses only the sign of the loss gradients.

The reference values $high_{n}^{j}$ and $low_{n}^{j}$ for accuracy term normalisation are computed as


(15) $$\begin{array}{cc} \;{\text{low}}_{n}^{j}=\text{Median}\left\{\text{NLL}\left[\text{w}\left({\text{s}}_{i+n}^{d}\right),\text{w}\left({\text{EM}}^{n}\text{D}\left({\text{s}}_{i}^{d},{\text{m}}_{i:i+n-1}^{d}\right)\right)\right]\right| & \\ \text{d}\in \left[0\ldots {\text{N}}_{v}\right)\wedge \text{i}\in \left[0\ldots \text{n}-3\right]\text{d}\in \left[0\ldots {\text{N}}_{v}\right)\wedge \text{i}\in \left[0\ldots \text{n}-3\right]\}, & \end{array}$$

(16) $$\begin{array}{cc} \;{\text{high}}_{n}^{j}=\text{Median}\left\{\text{NLL}\left[\text{w}\left({\text{s}}_{i+n}^{d}\right),\text{w}\left({\text{EM}}^{n}\text{D}\left({\text{s}}_{i}^{d},{\text{R}}^{m}\left(n\right)\right)\right)\right]\right| & \\ \text{d}\in \left[0\ldots {\text{N}}_{v}\right)\wedge \text{i}\in \left[0\ldots \text{n}-3\right]\text{d}\in \left[0\ldots {\text{N}}_{v}\right)\wedge \text{i}\in \left[0\ldots \text{n}-3\right]\} & \end{array}$$

where *N_v_* is the number of examples in the validation set (100 here), *d* indexes the example sequences in the dataset, *i* indexes the steps within an example sequence, *R^m^*(*n*) generates random manipulation sequences of length *n* (disregarding the restrictions used in data generation), and *Median* computes the median over its argument set. As for the range of *i*, recall that our example manipulation sequences are of length 3 (resulting in state sequences of length 4). We source example sequences of length *n* by selecting all sub-sequences indexed by *i* : *in* − 1 (inclusive) with *i*ı + *n*[0, 3 − *n*]. Hence for length *n* we find *4 - n* sequences per example.

These values give the range of loss values we can expect to see during successful plan search: $high_{n}^{j}$ gives the typical loss value for randomly initialised plans, while $low_{n}^{j}$ gives the typical residual loss expected for plans that in fact produce the goal state.

Epistemic uncertainty penalty *u* is calculated as


(17) $$\begin{array}{c} \text{u}=\sum_{i=1}^{n}\frac{{\text{e}}^{\beta \left(u_{i}-\gamma \right)}}{\text{n}}\; \end{array}\begin{array}{c} {\text{u}}_{i}=\frac{\text{diff}\left({\hat{\text{s}}}_{i}^{a},{\hat{\text{s}}}_{i}^{b}\right)-\mu_{i}^{v}}{\sigma_{i}^{v}}, \end{array}$$

where ${\hat{s}}_{i}^{j}$ is *net*_*j*_’s prediction of the cloth state at step *i* of the current plan, obtained by decoding the latent state ${\hat{c}}_{i}^{j}$ obtained when the current plan and state are fed into *net*_*j*_. Values $\mu_{i}^{v}$ and $\sigma_{i}^{v}$ are the mean and standard deviation of the difference between the two nets’ predictions at the *i^th^* state of their prediction sequences, computed over the validation set. This gives an estimate of the distribution of the difference between predictions for unseen examples drawn from the region of the state-action space covered by the training data. By normalising the difference, we avoid dependence on its absolute magnitude, which will differ for different network pairs. Parameters β and γ tune how prediction discrepancy impacts on the planning process. We set β to 5 and γ to 1.25 in our experiments. With these settings, planning is strongly averse to prediction discrepancies exceeding the expected discrepancy for plans in the known domain by over 1.25 standard deviations. The *diff* function quantifies the difference between two predictions. This requires some consideration, because whether or not a given network output can reasonably be interpreted as a prediction depends on whether the network inputs lie within (or near enough to) the known domain. For inputs outside the known domain, we should regard network output as nonsense. For this reason, we let the *diff* function compare its arguments simply as arrays of real values, instead of interpretating them as PMRs. We let *diff* whiten its arguments and compute their MSE.

Note that *u* is a penalty term, not a traditional probability measure. It is not intended to provide an interpretable quantitative measure of epistemic uncertainty, but to provide a gradient for steering the planning process toward plans that yield relatively low prediction discrepancy between the nets. Since prediction discrepancy is a proxy for epistemic uncertainty, this has the *effect* of avoiding epistemic uncertainty.

Implicit and explicit uncertainty avoidance affect plan search in subtly different ways. Only the latter specifically prevents the system from wandering into unknown state-action space territory. The implicit strategy may suffice to save the system from being thoroughly deceived by deceptive plans, but if it spends more update cycles in unknown regions (where gradients are unreliable), it will still produce worse outcomes than the explicit strategy.

Our experiments include single-net planning as a baseline. Single-net planning does not account for epistemic uncertainty (neither implicitly nor explicitly). The planning loss for single-net planning is $NLL\left(w\left(s^{*}\right),w\left({\hat{s}}_{n}\right)\right)$, with ${\hat{s}}_{n}$ being the predicted plan outcome.

## Experiments

We present three categories of results: estimation accuracy, prediction accuracy, and planning accuracy.

### Estimation

**FIGURE 6:**
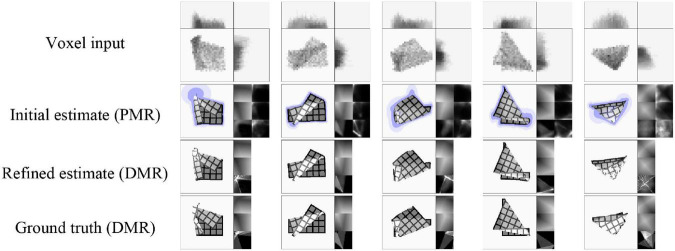
Representative examples of shape estimation and refinement (test set data). Each column represents one example. The last example shows a case where the z-ordering of the cloth layers is particularly difficult to infer from the voxel representation, leading to ambiguous z-ordering in the estimate.

First we evaluate shape estimation accuracy. This evaluation omits the first state (*s*_0_) of each data sequence. This state is always the flat default state, so including it would artificially inflate scores. Estimation accuracy is shown in Table 2. Scores indicate Euclidean distance between vertices’ estimated and actual positions, with the length of the cloth as unit. For the initial estimates (PMRs), scores are computed using the μ component. As noted above, refinement is intended to recover realism, and does not necessarily improve quantitative accuracy compared to the μ component of the initial estimate. The scores reveal some overfitting, and some errors in cloth layer ordering on the z-axis are observed, as well as instances of self-intersection in more complex cloth shapes (which is unsurprising, as the refinement routine does not perform collision checking). Self-intersection is not problematic for processing by the pEM*D net (self-intersection also occurs during pEM*D training due to noise augmentation). Examples of VtM estimates and their refinements are shown in Figure 6. We observe good approximation of global shape in the initial estimate, and recovery or fill-in of fine detail by refinement. Average time cost of mesh estimation is 3.7 milliseconds for the forward VtM pass generating the initial estimation and 0.57 s for refinement.

**TABLE 2:** Mesh estimation accuracy.

			Step			
Unit: Length of the cloth			1	2	3	All
Test (100 seq.)	PMR (μ-component)	mean (st.dev.) median	0.0142 (0.0201) 0.0116	0.0481 (0.0315) 0.0417	0.0800 (0.0383) 0.0781	0.0474 (0.0410) 0.0357
	DMR (refined)	mean (st.dev.) median	0.0137 (0.0198) 0.0114	0.0488 (0.0340) 0.0408	0.0833 (0.0410) 0.0831	0.0486 (0.0434) 0.0349
Train (100 seq.)	PMR (μ-component)	mean (st.dev.) median	0.0143 (0.00846) 0.0124	0.0418 (0.0242) 0.0354	0.0662 (0.0289) 0.0625	0.0408 (0.0308) 0.0331
	DMR (refined)	mean (st.dev.) median	0.0135 (0.00674) 0.0122	0.0420 (0.0255) 0.0342	0.0680 (0.0307) 0.0617	0.0411 (0.0323) 0.0315

To assess the quality of the aleatoric uncertainty estimations (σ component of the estimate), we divide errors by the corresponding σ values. Under perfect uncertainty estimation, this value should average to $\sqrt{2/\pi }\approx 0.798$ (the ratio between mean deviation and standard deviation for multivariate normal distributions). We obtain a median value of 0.770 for the test set, indicating that the σ component represents the actual uncertainty well, overestimating it slightly.

### Prediction

Next we evaluate the pEM*D net’s prediction ability. For purpose of dual-net planning we independently trained two identical networks. For the evaluation here and for single-net planning in the next section we use the net with the best validation score. We include two baselines:

• A1 (No VtM): Prediction without shape estimation (i.e., prediction using the initial state’s ground truth as input).
• A2 (Voxel-based): Prediction by an EM*D net trained to predict in voxel format [similar to previous work (Arnold and Yamazaki, 2019a)].

The voxel baseline requires some modification of the encoder and decoder modules, due to the different state input format. Following Arnold and Yamazaki (2019a), the encoder and decoder modules are composed of 6 3D-convolutional layers each, with 32, 32, 64, 128, 256, and 512 feature maps (order reversed for decoder). Kernel size is 3 × 3 × 3 throughout. We use striding to reduce resolution at each layer, with a stride of 2 for all dimensions in all layers, except for the z-dimension on the first layer (last layer in the decoder), which has a stride of 1. We also tested a voxel-based EM*D with densely connected layers (as the mesh-based system uses dense layers), but found convolution layers to perform better in the voxel version. The M module is identical between the voxel- and the mesh-based systems. Voxel representations are in the same format and resolution as used for VtM net input in the mesh-based system. As voxel representations are input to the planning network directly, the VtM net is not used in the voxel baseline.

**FIGURE 7:**
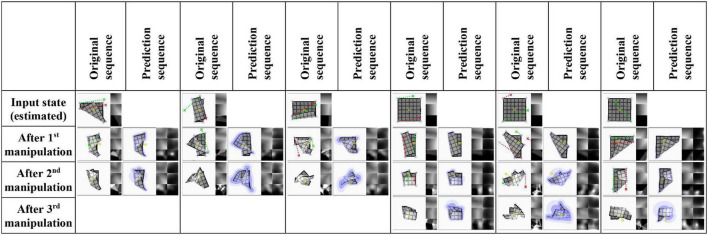
Example prediction results for sequences of two and three manipulations (main experiment, test set data). Input for prediction is an estimation of the original sequence’s first state and the sequence of manipulation inputs. Red and green dotted lines show manipulation trajectories. Lilac circles mark grasp point candidates. Yellow dotted lines on predictions indicate the predicted displacement of the cloth’s centre point. Trajectories that extend outside the viewport are wrapped around the border for visualisation purposes. The last example shows a case where the system fails to predict that the shape partially unfolds during the last manipulation.

Accuracies for prediction of the final outcome of manipulation sequences of length one, two, and three are given in Table 3. Evaluation is over the full test set (100 sequences) and 100 sequences from the training set. Prediction error increases with the length of the manipulation sequence, and some overfitting is again apparent. Score differences between the main experiment and baseline A1 (no VtM) are small, indicating that shape estimation is mostly sufficient to bring out the system’s predictive potential. Example predictions are shown in Figure 7. We observe that precision drops over the course of the sequence, but global shapes remain clearly recognisable for 3-step prediction. Increasing uncertainty over steps is expressed in increasing σ values over the course of the sequence (lilac shading).

**TABLE 3:** Prediction accuracy.

			Sequence length							
			1				2			3
Shape prediction accuracy Unit: Length of the cloth			Start point				Start point			Start point
			0	1	2	All	0	1	All	0/All
Test data (100 seq.)	Main	mean (st.dev.) median	0.0161 (0.00589) 0.01460	0.0451 (0.0244) 0.0381	0.0914 (0.0431) 0.0857	0.0509 (0.0423) 0.0362	0.0419 (0.0206) 0.0373	0.0837 (0.0364) 0.0753	0.0628 (0.0362) 0.0513	0.0809 (0.0347) 0.0737
	Baseline A1: No VtM	mean (st.dev.) median	0.0158 (0.00548) 0.0138	0.0412 (0.0214) 0.0349	0.0712 (0.0340) 0.0616	0.0428 (0.0325) 0.0325	0.0417 (0.0206) 0.0368	0.0811 (0.0355) 0.0719	0.0614 (0.0351) 0.0502	0.0810 (0.0347) 0.0738
Training data (100 seq.)	Main	mean (st.dev.) median	0.0163 (0.00726) 0.0137	0.0402 (0.0206) 0.0343	0.0773 (0.0391) 0.0673	0.0446 (0.0360) 0.0338	0.0389 (0.0188) 0.0332	0.0662 (0.0294) 0.0566	0.0525 (0.0282) 0.0453	0.0627 (0.0263) 0.0562
	Baseline A1: No VtM	mean (st.dev.) median	0.0162 (0.00728) 0.0136	0.0378 (0.0195) 0.0330	0.0571 (0.0274) 0.0491	0.0370 (0.0259) 0.0313	0.0386 (0.0186) 0.0332	0.0624 (0.0267) 0.0554	0.0505 (0.0259) 0.0442	0.0626 (0.0264) 0.0557
**Shape prediction accuracy** **Unit: mean voxel value difference**										
Test data (100 seq.)	Main	mean (st.dev.) median	**0.00858** (0.00284) **0.00833**	**0.0102** (0.00230) **0.00996**	**0.0131** (0.00289) **0.0126**	**0.0106** (0.00328) **0.0105**	**0.0102** (0.00232) **0.0101**	**0.0134** (0.00273) **0.0131**	**0.0118** (0.00301) **0.0114**	**0.0135** (0.00280) **0.0132**
	Baseline A2: Voxel-based	mean (st.dev.) median	0.00893 (0.00267) 0.00866	0.02069 (0.00607) 0.0202	0.0316 (0.00936) 0.0296	0.0204 (0.0114) 0.0192	0.0200 (0.00598) 0.0193	0.0326 (0.00891) 0.0329	0.0263 (0.00986) 0.0242	0.0325 (0.00885) 0.0330
Training data (100 seq.)	Main	mean (st.dev.) median	**0.00782** (0.00262) **0.00744**	**0.00959** (0.00223) **0.00934**	**0.0121** (0.00266) **0.0116**	**0.00983** (0.00306) **0.00962**	**0.00958** (0.00223) **0.00920**	**0.0121** (0.00261) **0.0116**	**0.0109** (0.00275) **0.0103**	**0.0120** (0.00258) **0.0115**
	Baseline A2: Voxel-based	mean (st.dev.) median	0.00911 (0.00279) 0.00866	0.0195 (0.00572) 0.0189	0.0272 (0.00607) 0.0270	0.0186 (0.00899) 0.0180	0.0187 (0.00537) 0.0177	0.0267 (0.00560) 0.0259	0.0227 (0.00678) 0.0220	0.0266 (0.00544) 0.0263

**Position prediction accuracy** **Unit: Length of the cloth**			**Sequence length**							
			**1**				**2**			**3**

Test data (100 seq.)	Main	mean (st.dev.) median	0.0295 (0.0352) 0.0174				0.0383 (0.0346) 0.0282			0.0505 (0.0407) 0.0353
	Baseline A1: No VtM	mean (st.dev.) median	0.0269 (0.0295) 0.0163				0.0379 (0.0349) 0.0259			0.0506 (0.0403) 0.0370
Training data (100 seq.)	Main	mean (st.dev.) median	0.0235 (0.0262) 0.0147				0.0214 (0.0175) 0.0189			0.0205 (0.0115) 0.0192
	Baseline A1: No VtM	mean (st.dev.) median	0.0164 (0.0114) 0.0138				0.0197 (0.0108) 0.0180			0.0201 (0.0114) 0.0180

Bold values indicate the best across all setups (main experiment and baselines), excluding baselines A1 and B1.

We evaluate aleatoric uncertainty estimates by dividing the error for each predicted vertex coordinate by the corresponding sigma value, and averaging the results. We obtain median values of 0.750, 0.785 and 0.861 for 1-, 2-, and 3-step prediction, respectively, indicating decent uncertainty estimation (recall that the ideal value is 0.798).

Table 3 also reports accuracy of the predicted cloth centre position shift for the main system configuration and the no-VtM baseline (A1). We see that position shift is predicted with decent accuracy, which means we can calculate the approximate position of the predicted shapes in the workspace. Although not used here, this functionality would be important in practical scenarios with workspace and arm reach limitations.

To evaluate baseline A2 (voxel-based), we need to compare shape prediction ability across shape representation formats. For this purpose, we convert predictions from the mesh-based main experiment into voxel format as follows. For each vertex *i*, we draw 1024 random samples from the multivariate normal distribution given for vertex *i* by the prediction. We bin the samples into voxels, and then divide the voxel values by 1024. This results in a voxel-format prediction for the single vertex, consisting of values $p_{xyz}^{i}$ quantifying the probability of vertex *i* falling in the voxel with indices *(x, y, z)*. We combine the single-vertex predictions into a full-cloth prediction by calculating for each voxel the probability that one or more vertices fall into it as


(18) $$\begin{array}{c} p_{xyz}=1-\prod_{i=0}^{n_{vertices}}\left(1-p_{xyz}^{i}\right). \end{array}$$

We now have occupancy probabilities for all voxels, which together constitute a cloth-shape prediction in voxel format. This format corresponds to the state format of the voxel version of the planning system, allowing for direct accuracy comparison. Results are given in Table 3. The error unit here is the mean voxel value difference. The ground truth is a binary voxel representation (1 for voxels occupied by the cloth, 0 for empty voxels). The best mean and median score for each sequence type is bolded. Mesh-based prediction outperforms voxel-based prediction by a good margin, despite the fact that the mesh-based predictions pass through two noisy format conversions in this evaluation. State input to both systems is the same, but recall that the mesh-based system takes grasp point input in geodesic coordinates. As there exists no geodesic space in the voxel-based system, it takes grasp points in Cartesian coordinates.

### Planning (simulation)

Next we evaluate planning performance. In addition to the main system, we evaluate five baselines to assess the contributions of specific aspects.

• B1: No VtM. Performs planning on basis of ground truth meshes instead of estimated meshes. This baseline measures planning performance with perfect perception, allowing assessment of how well the VtM net performs in the context of the full planning system.
• B2: Single-net planning. Isolates the contribution of our dual-net epistemic uncertainty avoidance strategy.
• B3: Dual-net planning with implicit epistemic uncertainty avoidance. Isolates the contribution of the explicit epistemic uncertainty penalty *u*. Obtained by setting α to 0 in Equation 14.
• B4: Voxel-based dual-net planning. For comparison with the voxel-based approach in Arnold and Yamazaki (2019a), we perform planning with EM*D nets operating on voxel representations directly. This baseline adds dual-net planning functionality to the voxel version.
• B5: Voxel-based single-net planning. This baseline closely approximates (Arnold and Yamazaki, 2019a).

**FIGURE 8:**
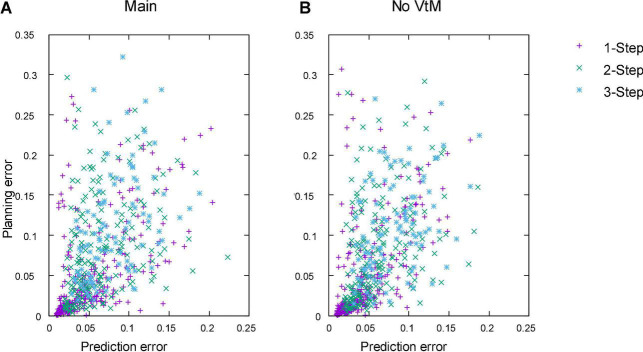
Planning accuracy plotted against prediction accuracy for all 1-, 2-, and 3-step sequences in the test set. **(A)** Main experiment. **(B)** Baseline B1 (No VtM). Error unit: length of the cloth. For predictions, errors are calculated for the μ component of the prediction. Unit is the length of the side of the cloth.

**FIGURE 9:**
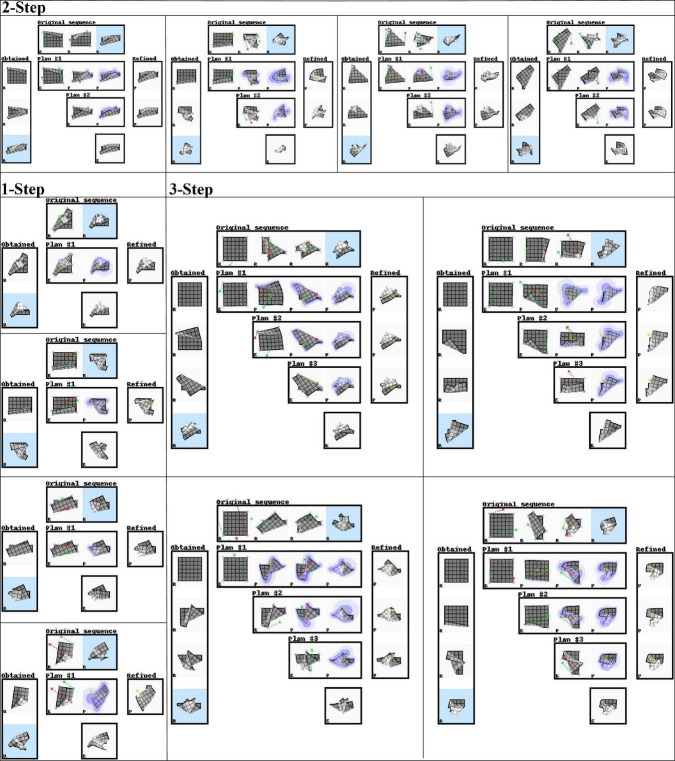
Representative examples of planning and execution sessions (test set data, dual-net planning). In each panel, the top row shows the original manipulation sequence. The system only sees its estimate of the current state and the final state of the original sequence (i.e., the goal state, marked with light blue background). The left column shows the sequence of cloth shapes obtained over the course of the session. The final outcome is marked with a light blue background. Rows marked “Plan #*i*” show the *i^th^* plan generated in the session. Each plan starts from an estimate (DMR) of the current shape, generated through the VtM net and refinement with subsequent shapes being predictions (PMRs) generated by the pEM*D net. Under the last plan, we see the system’s estimation of the obtained outcome. The right column shows the result of applying the refinement procedure to the predicted outcome of each plan. These are added for illustration, and not used by the system. They represent a plausible deterministic shape drawn from the probabilistic prediction of each plan’s outcome. All shapes are marked in their bottom left corner to indicate the shape type: R = real, E = estimation, P = prediction. Red and green dotted lines show manipulation trajectories. Lilac circles mark grasp point candidates.

We evaluate planning ability on all 100 examples from the test set and 100 examples from the training set. We use all sequences of length one to three that can be sourced from this set of examples. The same sequences are used for each experiment. Table 4 shows accuracy of the outcomes of interleaved planning and execution measured as the Euclidean distance between goal and outcome for each cloth vertex, with the length of the side of the cloth as unit. The best mean and median score for each sequence type is bolded (disregarding baseline B1 because it is advantaged by perfect perception). Our planning conditions aim to optimise oriented shape, while placing no constraints on the position of the obtained shape in space. Hence for evaluation, we align the outcome and goal shapes in space. We do this by computing offset vectors (the difference between a vertex’s position in the goal shape and the outcome shape) for all vertices, and subtracting the average offset vector from all vertices. Scatter plots in Figure 8 show the scores for all examples from the test set for the main experiment and baseline B1, plotting planning accuracy against prediction accuracy as evaluated in the previous section. Figure 9 shows examples of planning and execution sessions. Note that many goal shapes can be produced through more than one manipulation sequence. In a number of the multi-step examples shown in Figure 9, we see that the system invented manipulation sequences that differ from the original sequence, yet closely approximate its outcome. Parallel instances of the planning process, as well as repeated runs of the plan generation process, can produce different valid plans.

**TABLE 4:** Planning accuracy.

			Sequence length							
			1				2			3
			Start point				Start point			Start point
Unit: Length of the cloth			*0*	*1*	*2*	*All*	*0*	*1*	*All*	*0/All*
Test data (100 seq.)	*Main*	mean (st.dev) median	0.0291 (0.0527) 0.00566	0.0411 (0.0499) **0.0223**	0.0783 (0.0637) 0.0591	0.0495 (0.0596) **0.0232**	**0.0725** (0.0680) **0.0453**	**0.0913** (0.0596) **0.0819**	**0.0819** (0.0646) **0.0643**	**0.109** (0.0652) **0.0969**
	*Baseline B1*: *no VtM*	mean (st.dev) median	0.0288 (0.0591) 0.00565	0.0395 (0.0501) 0.0193	0.0718 (0.0637) 0.0488	0.0467 (0.0607) 0.0185	0.0678 (0.0596) 0.0495	0.0905 (0.0639) 0.0803	0.0792 (0.0628) 0.0619	0.109 (0.0575) 0.102
	*Baseline B2*: *single-net*	mean (st.dev) median	0.0333 (0.0640) 0.00654	**0.0402** (0.0425) 0.0259	0.0778 (0.0629) **0.0550**	0.0504 (0.0606) 0.0259	0.0809 (0.0611) 0.0640	0.0986 (0.0595) 0.0917	0.0898 (0.0610) 0.0819	0.126 (0.0712) 0.123
	*Baseline B3*: *dual-net without* *uncertainty loss*	mean (st.dev) median	0.0314 (0.0577) **0.00550**	0.0408 (0.0485) 0.0246	**0.0677** (0.0510) 0.0588	**0.0466** (0.0547) 0.0252	0.0813 (0.0736) 0.0598	0.0985 (0.0602) 0.0844	0.0899 (0.0678) 0.0726	0.121 (0.0665) 0.124
	*Baseline B4*: *Voxel / dual*	mean (st.dev) median	**0.0278** (0.0336) 0.0140	0.0635 (0.0531) 0.0481	0.0915 (0.0669) 0.0689	0.0609 (0.0590) 0.0370	0.112 (0.0764) 0.0887	0.132 (0.0663) 0.127	0.122 (0.0722) 0.114	0.169 (0.0599) 0.181
	*Baseline B5*: *Voxel / single*	mean (st.dev) median	0.0319 (0.0502) 0.0129	0.0790 (0.0668) 0.0546	0.0942 (0.0655) 0.0782	0.0683 (0.0668) 0.0445	0.132 (0.0752) 0.127	0.162 (0.0712) 0.168	0.147 (0.0748) 0.156	0.192 (0.0590) 0.205
Training data (100 seq.)	*Main*	mean (st.dev) median	0.0384 (0.0605) **0.00625**	0.0407 (0.0488) 0.0251	0.0718 (0.0640) **0.0423**	0.0503 (0.0601) 0.0267	**0.0714** (0.0529) **0.0481**	0.0902 (0.0583) 0.0770	**0.0808** (0.0565) **0.0636**	**0.119** (0.0612) **0.115**
	*Baseline B1*: *no VtM*	mean (st.dev) median	0.0417 (0.0743) 0.00639	0.0343 (0.0397) 0.0186	0.0631 (0.0618) 0.0334	0.0464 (0.0615) 0.0203	0.0731 (0.0614) 0.0462	0.0867 (0.0659) 0.0657	0.0799 (0.0641) 0.0582	0.109 (0.0570) 0.0992
	*Baseline B2*: *single-net*	mean (st.dev) median	0.0426 (0.0654) 0.00709	**0.0345** (0.0379) **0.0191**	**0.0687** (0.0572) 0.0491	**0.0486** (0.0566) **0.0248**	0.0925 (0.0692) 0.0867	0.0958 (0.0655) 0.0853	0.0942 (0.0674) 0.0862	0.127 (0.0667) 0.118
	*Baseline B3*: *dual-net without* *uncertainty loss*	mean (st.dev) median	0.0427 (0.0650) 0.00717	0.0372 (0.0449) 0.0212	0.0694 (0.0595) 0.0453	0.0495 (0.0588) 0.0251	0.0881 (0.0692) 0.0741	**0.0834** (0.0532) **0.0725**	0.0857 (0.0617) 0.0725	0.131 (0.0682) 0.129
	*Baseline B4*: *Voxel / dual*	mean (st.dev) median	**0.0383** (0.0446) 0.0166	0.0622 (0.0518) 0.0475	0.0876 (0.0673) 0.0652	0.0627 (0.0589) 0.0420	0.113 (0.0679) 0.105	0.132 (0.0676) 0.133	0.123 (0.0684) 0.119	0.169 (0.0574) 0.172
	*Baseline B5*: *Voxel / single*	mean (st.dev) median	0.0514 (0.0698) 0.0179	0.0782 (0.0741) 0.0503	0.0835 (0.0676) 0.0593	0.0711 (0.0719) 0.0435	0.155 (0.0813) 0.159	0.154 (0.0714) 0.158	0.155 (0.0765) 0.159	0.202 (0.0543) 0.207
**Unit: mean voxel value difference**										
Test data	Main	mean (st.dev) median	**0.00282** (0.00347) **0.00150**	**0.00516** (0.00427) **0.00394**	**0.00885** (0.00501) **0.00793**	**0.00561** (0.00496) **0.00412**	**0.00783** (0.00511) **0.00662**	**0.0103** (0.00484) **0.00964**	**0.00907** (0.00512) **0.00836**	**0.0119** (0.00507) **0.0116**
	Baseline B4: Voxel / dual	mean (st.dev) median	0.00768 (0.00722) 0.00504	0.0182 (0.00985) 0.0166	0.0254 (0.0117) 0.0243	0.0171 (0.0122) 0.0146	0.0239 (0.0114) 0.0222	0.0308 (0.0112) 0.0305	0.0273 (0.0118) 0.0264	0.0336 (0.00866) 0.0332
	Baseline B5: Voxel / single	mean (st.dev) median	0.00818 (0.00810) 0.00455	0.0211 (0.0117) 0.0203	0.0269 (0.0127) 0.0249	0.0187 (0.0135) 0.0170	0.0282 (0.0127) 0.0289	0.0361 (0.0134) 0.0380	0.0322 (0.0136) 0.0319	0.0412 (0.0122) 0.0420
Training data	Main	mean (st.dev) median	**0.00323** (0.00386) **0.00146**	**0.00523** (0.00407) **0.00421**	**0.008302** (0.00545) **0.00681**	**0.00558** (0.00498) **0.00406**	**0.00825** (0.00457) **0.00717**	**0.0104** (0.00477) **0.0103**	**0.00932** (0.00479) **0.00845**	**0.0121** (0.00478) **0.0120**
	Baseline B4: Voxel / dual	mean (st.dev) median	0.00894 (0.00767) 0.00650	0.0185 (0.00937) 0.0167	0.0251 (0.0122) 0.0224	0.0175 (0.0119) 0.0160	0.0237 (0.00984) 0.0230	0.0306 (0.0111) 0.0302	0.0272 (0.0111) 0.0264	0.0338 (0.00958) 0.0341
	Baseline B5: Voxel / single	mean (st.dev) median	0.0105 (0.00970) 0.00641	0.0211 (0.0129) 0.0180	0.0241 (0.0126) 0.0227	0.0186 (0.0132) 0.0165	0.0314 (0.0133) 0.0302	0.0355 (0.0131) 0.0340	0.0335 (0.0133) 0.0322	0.0424 (0.0114) 0.0441

Bold values indicate the best across all setups (main experiment and baselines), excluding baselines A1 and B1.

Planning on ground truths instead of estimates (baseline B1) produces slightly better scores for most cases, but the difference is modest. This indicates that the quality of the VtM net’s mesh estimates is sufficient for use in this planning system, at least when operating on simulation data.

Dual-net planning outperforms single-net planning (baseline B2), which shows the effectiveness of epistemic uncertainty avoidance for planning on our incompletely sampled domain. Furthermore we see that the explicit strategy improves performance compared to the implicit strategy (baseline B3).

Regardless of the shape representation format used by the planning system, the result of a planning session is a mesh representation from the simulation environment. Hence unlike predictions, planning outcomes can be compared directly in mesh form between the mesh- and voxel-based versions of the system. We see that the mesh-based system obtains better accuracy for both dual- (main vs. B4) and single-net (B2 vs. B5) planning. However, this comparison is somewhat unfair: the voxel-based system is set up to optimise accuracy measured in voxel format. We convert outcomes to voxel form, and report voxel-based accuracy in Table 4. Even with accuracy measured in voxel format, mesh-based planning outperforms voxel-based planning.

Planning times are shown in Table 5. Time cost for the main configuration and baselines were measured on a single GeForce RTX 2080 Ti GPU, with the system implemented in TensorFlow (Abadi et al., 2015). Additionally, time cost for a more optimised JAX (Bradbury et al., 2018) implementation of the main configuration was measured on a single GeForce RTX3090 GPU. As is to be expected, planning times are longer for dual-net planning than single-net planning. Calculation of the epistemic uncertainty penalty *u* requires decoding of intermediate states, which adds substantial computational cost. However, planning times remain on the order of seconds for the mesh-based system. With regards to the voxel versions, planning times are longer than those reported in Arnold and Yamazaki (2019a). This is because we increased the number of parallel search strains to match the mesh version, for fairness of the accuracy comparison. Planning times do indicate a computational cost advantage of the mesh version.

**TABLE 5:** Planning time cost.

Time cost (seconds)	Implementation and GPU	Plan length		
		*1*	*2*	*3*
Main Mesh / dual	TensorFlow 1 / RTX 2080 Ti	4.76s	9.11s	14.7s
Baseline B2: Mesh / single		1.68s	2.98s	4.71s
Baseline B4: Voxel / dual		88.3s	192s	335s
Baseline B5: Voxel / single		22.2s	33.9s	45.9s
Main Mesh / Dual	JAX / RTX 3090	2.65s	4.44s	6.49s

### Failure modes

The existence of large-error outliers is evident in Figure 8. We observe a few failure modes. One originates in the grasp point detection routine. Sometimes a corner of the refined VtM estimate is less pronounced than in the ground truth and fails to be detected as graspable. Consequently, plan search fails to consider the grasp point, potentially making it impossible to find the optimal manipulation. We also observe errors in the z-ordering of cloth layers in shapes with multiple overlapping folds. For example, when the target shape folds the cloth in two over its x axis and then over its y axis, the generated plan may sometimes fold in reverse order. This may be due to alternative fold orders representing local minima in the plan search space. Loss definitions that accentuate erroneous z-ordering should help to ameliorate this. Finally, the mesh estimation routine sometimes estimates incorrect z-ordering. Some shapes become ambiguous in voxel representation. When such a shape is the product of a preceding manipulation by the system, it may still be disambiguated during refinement (which in this case is initialised with the predicted outcome of the preceding manipulation). However, when a manipulation session starts from such a shape, failure is likely.

### Planning (hardware)

We integrated the planning system with a dual-handed robot platform (HIRO, Kawada Robotics), and performed qualitative experiments on real cloth. There are significant differences between the simulated cloth and real cloth, in particular in thickness, friction with the work surface, and elasticity. We manually selected five examples for which we expect the manipulation sequence to produce similar results on our simulated and real cloth. The mesh of the input state is estimated from a voxelised point cloud obtained using a Kinect depth camera mounted on the robot’s head, providing a near-top-down perspective on the cloth. We performed interleaved planning and execution following the same procedure as in the simulation experiments. To grasp the cloth, we let one finger of the gripper slide underneath it at the grasp position, and then close the gripper. The closure direction aligns with the z-axis. Hence, only the x and y coordinates of the grasp point are required.

**FIGURE 10:**
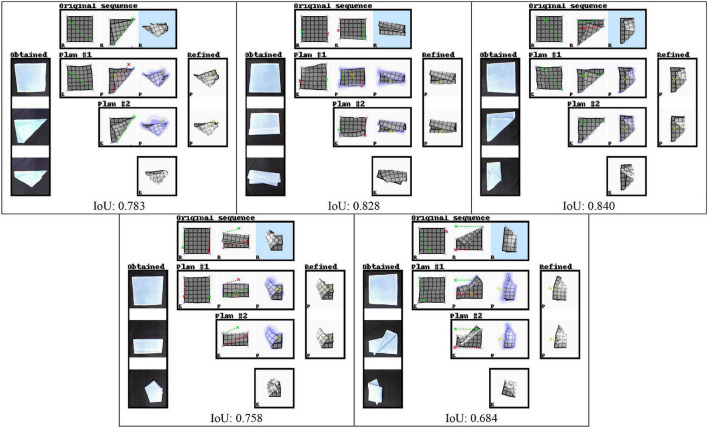
Two-step manipulation sequences planned by the system, performed by a dual-handed robot on real cloth. See Figure 9 for the figure format. The right-most column in each example shows the sequence of actually obtained physical cloth shapes. Input states for plan generation are obtained by shape estimation on voxelised point cloud data of the real cloth. Real cloth shapes are captured at a slight angle due to the camera placement. Plans were rotated by multiples of 90° degrees around the z-axis in order to accommodate limitations of the robot’s range, and images of real cloth shapes are rotated accordingly. Scores are Intersection-over-Union scores over mask images computed for the goal and outcome, indicating the similarity of the top-down silhouettes of goal and outcome shapes, with 1.0 corresponding to a perfect match.

Figure 10 shows results in the same format as Figure 9, and still frames of one example case are shown in Figure 1. We observe that most outcomes clearly resemble the goal states. For many manipulations seen here, close inspection reveals that the trajectories slightly overshoot the optimal release point, leading to folds that are slightly deeper than intended. This is due to the difference in elasticity between the simulated and real cloth. The simulated cloth stretches somewhat under its own weight when lifted, causing it to rebound slightly upon release. The planning system, anticipating this behaviour, places the release points slightly beyond the grasped points’ intended final positions. The real cloth, however, stabilises without rebounding, resulting in the slight overshoot seen here.

We quantify similarity of the outcomes to the goals using IoU (Intersection-over-Union) scores computed over top-down mask images (i.e., silhouettes) of the goal and outcome. Scores are shown in the figure. Shape estimation accuracy on real cloth is sufficient on the initial and intermediate shapes to allow for effective planning, but estimation on the final outcome is seen to be challenging in cases where many layers of cloth overlap. This is partly due to the difference in thickness between the simulated and real cloth, which causes increasing divergence between the voxel representations of simulated and real cloth as the number of stacked layers increases.

## Discussion

Our experimental results on simulated cloth confirm that accuracy is significantly improved by the use of mesh representations and dual-net planning with an explicit epistemic uncertainty penalty. The advantage of mesh representation holds up when we evaluate outcomes in voxel form, reflecting the fact that accuracy in mesh format implies accuracy in voxel format, but not vice-versa.

The present work deviates from the trend of letting systems encode raw sensor data (pixels/voxels) into latent representations (Arnold and Yamazaki, 2019a; Hoque et al., 2020; Wahlström et al., 2015; Yan et al., 2020). Central to this trend is the idea that training will discover the data’s deep structure. Our results suggest this may be optimistic. The mesh and voxel versions of the system operate on identical voxel input, and generate latent representations of identical dimensionality, yet show a substantial performance gap. This suggests that the training procedure (a fairly typical deep learning training procedure) is not actually capable of discovering the data’s deep structure by itself.

The advantage of setting goal states in mesh format also bears emphasising. When we consider tasks such as garment folding or knot-tying, we aim not just for visual similarity from a given viewpoint, but for structural similarity to the goal state. This is hard to achieve when the system does not represent the object’s structure in a form that allows for comparison to a structured goal representation.

We find that in numerous cases, planning outcomes more closely resemble the goal state than the system’s own predictions for the generated plan. This may seem counterintuitive, but should not be surprising. In principle, close resemblance between the goal state and the prediction for the optimal action sequence is no requirement for finding the optimal action sequence. What is required is merely that the prediction for the optimal action sequence better approximate the goal than the predictions for non-optimal action sequences. This allows significant leeway in prediction accuracy. The use of explicit aleatoric uncertainty in the mesh format helps the system exploit this leeway. For parts of the state that the system cannot predict with precision, it indicates lack of confidence with elevated σ values. This reduces the impact of these parts on the planning loss. Parts that are predicted with relatively high confidence thus dominate plan search. When the relatively predictable parts of the deformation process sufficiently constrain action search, near-optimal actions can be found despite significant uncertainty about other parts. If the action is correct and the deformation dynamics are consistent, the hard-to-predict parts will fall into place when the action is executed. Hence the combination of explicit aleatoric uncertainty and backpropagation-based planning with a likelihood planning loss allow for effective planning even in presence of substantial prediction uncertainty.

The effectiveness of the dual-net approach raises the question whether additional network instances could further boost performance. We expect the utility of additional instances to be marginal. The introduction of the second net adds functionality by providing a gradient toward the ROI. The utility of additional nets would be limited to reducing the noisiness of this gradient. With regard to deception avoidance, the dual-net approach is based on the assumption that deceptive plans appear at more or less random points in the input space, and that these points differ for independently trained nets. If this assumption holds, then very few plans will deceive two nets at once, making the utility of additional nets for deception avoidance negligible.

Our dataset includes complex shapes of limited practical use. The choice not to constrain the repertoire to basic, common cases is motivated by a pursuit of generalised affinity for cloth manipulation. Generality implies that the system can generate plans for both unseen novel cases as well as cases of no practical use. The alternative is to assume that we can imagine and account for all the situations a robotic system will find itself in, which may be a strong assumption to make when a robot is supposed to function flexibly in an uncontrolled environment.

## Conclusion and future work

We presented a system for cloth manipulation planning, building on the system proposed in Arnold and Yamazaki (2019a). We adopted mesh representations to eliminate much of the ambiguity that hampers voxel-based planning. Mesh representations were obtained by introducing a neural network-based shape estimation routine into the system. We found that this significantly improves accuracy for both prediction and planning. We also addressed the problem of planning with incomplete domain knowledge, introducing a dual-network planning strategy that uses prediction discrepancy between networks as a proxy of epistemic uncertainty in order to avoid epistemic uncertainty. Finally, the need to work with discrete sets of graspable points was addressed by incorporating grasp point detection. These improvements make significant strides toward practical applicability. With regard to time cost, dual-net planning is more demanding than single-net planning, but the switch to comparatively lightweight representations offsets the additional cost. Planning times on the order of a few seconds are maintained.

Various avenues for further development remain. At present, planning is closed-loop at the granularity of full manipulation steps. Correction of trajectories mid-manipulation can be achieved by applying many of the same concepts at a finer timescale. We pursue these ideas in parallel work (Tanaka et al., 2021). We used a simple square cloth here. No part of the system relies on this topology, but performance on alternative topologies remains to be assessed. Discrepancies in material properties between simulated and real cloth are an obstacle to real-world generalisation, as we observed in our hardware experiments. Settings where we cannot assume accurate prior knowledge of fabric properties (such as household support settings) require a structured way of handling material variation. We believe this is an important open issue in cloth manipulation research. In ongoing work, we are developing functionality for estimating material properties on the fly during manipulation and using these estimates to improve manipulation trajectories (Arnold and Yamazaki, 2022). Other work in this Research Topic addresses this issue by means of parametric biases (Kawaharazuka et al., 2022).

Voxelisation of the shape input to the VtM net may cause shape ambiguity as discussed in Section 6.4, and limits fidelity to some extent. Using undiscretised point cloud input may further improve mesh estimation accuracy while retaining the generalisation properties of voxel representations. To this end, we are experimenting with estimation architectures based on PointNet (Qi et al., 2017). Finally, our planning evaluation experiments assumed plan length to be given, which is impractical. Planning with variable plan lengths can be achieved using a planning loss that compares predicted intermediate states against the goal state.

## Data availability statement

The raw data supporting the conclusions of this article will be made available by the authors, without undue reservation.

## Author contributions

SA, KY, and DT contributed to conception and design of the study. SA implemented the planning system and integration with simulation environment, performed simulation experiments, and wrote the manuscript. DT integrated the system with robot hardware. DT and SA performed the hardware experiments. All authors contributed to the article and approved the submitted version.

## Footnotes

1 White box attack: an attack where the adversary has full access to the NN.
